# The Effects of a Healthy Lifestyle on Depressive Symptoms in Older Chinese Adults: The Mediating Role of Psychological Resilience

**DOI:** 10.7759/cureus.57258

**Published:** 2024-03-30

**Authors:** Ailing Duan, Hang Zhao, Chunmin Zhou

**Affiliations:** 1 Public Health, Chongqing Medical University, Chongqing, CHN; 2 Public Health, Chongqing Medical University, Chonqing, CHN

**Keywords:** mediating effect, depression symptoms, psychological resilience, healthy lifestyles, older adults

## Abstract

Objectives

This study aimed to validate the interrelationships and potential pathways of influence between healthy lifestyles, psychological resilience, and depressive symptoms in the Chinese elderly population.

Methods

We utilized data from the Chinese Elderly Health Influential Factors Tracking Survey 2018 and included 9448 samples for the study after screening according to the qualifying conditions. The interrelationships among healthy lifestyles, psychological resilience and depressive symptoms were analyzed using stepwise regression, and the robustness of mediation effects was assessed using Sobel and Bootstrap test.

Results

Among Chinese older adults, healthy lifestyles were negatively associated with depressive symptoms (β = -0.310, 95% CI: -0.405, -0.215), positively associated with psychological resilience (β = 0.137, 95% CI:0.071, 0.023), and psychological resilience was negatively associated with depressive symptoms (β = -1.014, 95% CI: -1.037, -0.990).

Conclusions

Psychological resilience partially mediated the association between healthy lifestyles and depressive symptoms, with the mediating effect accounting for 44.8% of the total effect. Our study contributes to the understanding of the relationship between healthy lifestyles and depressive symptoms in the elderly population and emphasizes the important role of psychological resilience. It is recommended that the government and policymakers improve depressive symptoms among older adults through comprehensive measures such as promoting healthy lifestyles and education, providing psychological support services, and creating a favorable environment.

## Introduction

Population aging has emerged as a worldwide phenomenon concomitant with socio-economic development and extended human longevity [1]. China, in particular, is currently undergoing a rapid aging process. As of 2020, the mainland region already harbors a total of 264 million elderly individuals aged 60 years and older, constituting 18.7% of the overall population [2]. With population aging, depression, as a prevalent mental disorder among the elderly population, has become a global public health issue [3]. Surveys have shown that the overall prevalence of depression in older adults is 35.1% globally [4], and the prevalence of depression in older adults in China is 23.6% [5]. The current elevated prevalence of depression poses substantial consequences for the physical and mental well-being of older individuals, manifesting in diminished quality of life, cognitive decline, and adverse health outcomes [6]. Research indicates that both severely and mildly depressed older adults experience a lower quality of life compared to their non-depressed counterparts [7]. Moreover, depression is intricately linked to an increased risk of Alzheimer's disease [8]. Alarmingly, suicide rates among older adults are twice as elevated compared to the general population [9]. Beyond the immediate health implications, depression in older adults imposes a considerable economic burden on both society and families [10]. Consequently, addressing the challenges posed by global aging and enhancing the quality of life for older adults necessitates a thorough investigation into the influences and potential pathways associated with depressive symptoms in this demographic.

The World Health Organization defines healthy lifestyles as diminishing the risk of serious illness or premature death [11]. Physical activity, a healthy diet, and avoidance of smoking and alcohol have been identified as the primary components of healthy lifestyles, according to HWO recommendations. There are pieces of evidence of a correlation between healthy lifestyles and depressive symptoms, particularly in older adults. Several scholars have explored the relationship between different lifestyles and depressive symptoms among the elderly. Research indicates that individuals engaging in regular physical activity exhibit higher aerobic fitness, which is associated with a diminished risk of depressive symptoms in older adults [12]. Concurrently, adopting a healthy diet, characterized by the consumption of vegetables, fruits and antioxidant-rich foods, has been identified as a protective factor against depressive symptoms in this demographic [13]. Furthermore, smoking and alcohol consumption demonstrate significant correlations with depressive symptoms in older adults [14, 15]. For instance, compared to those who continue to smoke, those who quit smoking have their neurological functioning restored [16] and thus display a more positive mood [17], while problematic alcohol consumption heightens the risk of depressive symptoms in older adults [18]. Despite evidence supporting the positive impact of healthy lifestyles on depressive symptoms in older adults, previous studies have focused on single healthy lifestyle behavior. Fewer investigations have explored the effects of integrated healthy lifestyles on depressive symptoms. In addition, most of the available evidence comes from studies in developed countries, and there is a lack of evidence from developing countries. It is challenging to apply evidence from developed countries directly to developing countries due to differences in economic development, genetic factors, living environments, and sociocultural contexts. Given that China is the world's largest developing country and has the largest aging population [19], the relationship between healthy lifestyles and depressive symptoms in Chinese older adults is crucial. It is therefore imperative to explore the effects of integrated healthy lifestyles on depressive symptoms in older adults in the context of aging in China.

Psychological resilience is the capacity or trait to adapt to a stressor successfully and remain psychologically healthy in the face of it [20]. Numerous studies have demonstrated that psychological resilience has a substantial correlation with depressive symptoms [21-23]. Psychological resilience is a protective factor against depressive symptoms and plays a positive role in promoting mental health [24]. Among older adults, psychological resilience demonstrates a close association with amygdala function, where heightened amygdala function assumes a pivotal role in depressive symptoms among this demographic [25]. Meanwhile, psychological resilience has also been proven to play a connective role in multiple factors and depressive symptoms. For instance, a study conducted in Jinan, China, investigated the relationship between loneliness, psychological resilience, social support, and depressive symptoms among elderly individuals in nursing homes. The findings showed that interventions such as improving psychological resilience and social support may help break the link between loneliness and depressive symptoms in elderly nursing home residents [26]. Psychological resilience has also been shown to be associated with positive social engagement. It mediates the link between social engagement in older adult depressive symptoms and alleviates stress in widowed older adults [27]. Despite these insights, scant attention has been devoted to exploring the relationship between healthy lifestyles and psychological resilience. It remains unclear whether psychological resilience would play a mediating role between healthy lifestyles and depressive symptoms.

According to the above literature and theories, the relationship between healthy lifestyles and depressive symptoms is to be further clarified, and the mediating role of psychological resilience in the relationship between healthy lifestyles and depressive symptoms has not been validated among older adults in the context of aging in China. Thus, this study aims to investigate the associations among healthy lifestyles, psychological resilience, and depressive symptoms in Chinese older adults, utilizing data from the China Lingering Health Influence Survey (CLHLS). Based on previous research, four hypotheses were proposed: hypothesis one is that healthy lifestyles are negatively associated with depressive symptoms. Hypothesis two is that healthy lifestyles are positively associated with psychological resilience. Hypothesis three is that psychological resilience is negatively related to depressive symptoms. Hypothesis four is that psychological resilience mediates the relationship between healthy lifestyles and depressive symptoms.

## Materials and methods

Participants

This study aimed to validate the interrelationships and potential pathways of influence between healthy lifestyles, psychological resilience, and depressive symptoms in the Chinese elderly population. Stepwise regression method and Sobel and Bootstrap tests were used to verify their relationships. In addition, no ethical review was required as publicly available data was used in the study. The data for this study came from the Chinese Longitudinal Healthy Longevity Survey (CLHLS), which is the China Geriatric Health Survey conducted by the Chinese Center for Disease Control and Prevention in collaboration with the Center for Healthy Aging and Development at the National Institute of Development Studies of Peking University. The CLHLS is a comprehensive nationwide survey spanning 22 provinces, municipalities, and autonomous regions, encompassing over 500 sample sites and individuals aged 65 years and older. Employing a randomized household survey methodology, the CLHLS collects information on participants' basic details, dietary habits, lifestyle, behaviors, and illnesses. Informed consent was obtained from all participants or their legal representatives, and trained health workers administered the survey using an internationally standardized questionnaire [28]. The analysis used data from the 2018 CLHLS, which included a total of 15,874 samples. Inclusion criteria for this study included those aged 65 and older and those without missing key variables, and exclusion criteria included those with missing key variables. The final inclusion was 9448 samples after excluding 6426 samples.

Dependent variable: healthy lifestyles

Referring to the definitions of healthy lifestyles in existing studies [29-33] and combining the information collected by CLHLS, healthy lifestyles in our study included non-smoking, non-alcohol consumption, engaging in appropriate physical activity, and maintaining dietary diversity. Smoking, drinking, and physical activity are all binary variables, with 0 being "no" and 1 being "yes". Dietary diversity comprises diets inclusive of fruits, vegetables, meat, fish, eggs, soy products, nuts, milk, and tea. The Dietary Diversity Score (DDS) is the sum of the scores for all items, ranging from 0 to 9, with higher scores indicating greater dietary diversity. Having a good DDS (7-9 points) was scored as 1; otherwise, it was scored as 0 [34]. Ultimately, the scores for smoking, alcohol consumption, physical activity, and dietary diversity are aggregated to establish an overall healthy lifestyles score, ranging from 0 to 4, with higher scores indicative of healthier lifestyles.

Mediating variable: psychological resilience

Building on previous relevant research [35, 36], we used the following five questions to measure psychological resilience: (1) Are you able to think clearly no matter what happens?; (2) Do you have a say in your affairs?; (3) Do you feel that you are getting more and more useless as you get older and that it is very difficult for you to do anything?; (4) Do you feel nervous and scared?; (5) Do you feel lonely? Two forward problems were recorded by converting them to reverse problems and scored from 1 to 5 points each. A score of 1 means "always", 2 means "often", 3 means "sometimes", 4 means "rarely", and 5 means "never". The total score ranges from 5 to 25, with higher scores representing better mental resilience. This elasticity scale has been shown to have good reliability with a Cronbach's alpha coefficient of 0.89 [37].

Dependent variable: depressive symptoms

The Chinese version of the Center for Epidemiologic Studies Depression Scale (CES-D-10) was originally used to assess the level of depressive symptoms in older adults. The scale has been widely used in different populations with good reliability and validity [38, 39] and has also been well-validated in measuring depressive symptoms in older adults [40, 41]. Comprising 10 entries, including two positively and eight negatively rated, the scale initially featured two positive ratings, which were subsequently reversed to negative. Each entry is assigned a score on a four-point scale: 0 for "rarely" or "never", 1 for "sometimes", 2 for "often", and 3 for "always". Scores on the scale range from 0 to 30, with a total score of ≥10 indicating the presence of depressive symptoms. Elevated scores correspond to more severe depressive symptoms.

Covariates

We considered several potential confounders, including age, gender, ethnicity, household registration, marriage, years of education, residential arrangement, socioeconomic status, and self-rated health. Both age and years of education were treated as continuous variables. Gender (female and male), ethnicity (Han and Non-Han), household registration (rural and urban), marriage (married and unmarried, widowed, divorced, or separated), and living arrangement (living alone and not living alone) were represented as dichotomous variables. Socioeconomic status (poor, relatively poor, common, relatively rich, rich) and self-reported health (very bad, relatively bad, normal, relatively good, very good) were the five categorical variables.

Statistical analysis

**Figure 1:**
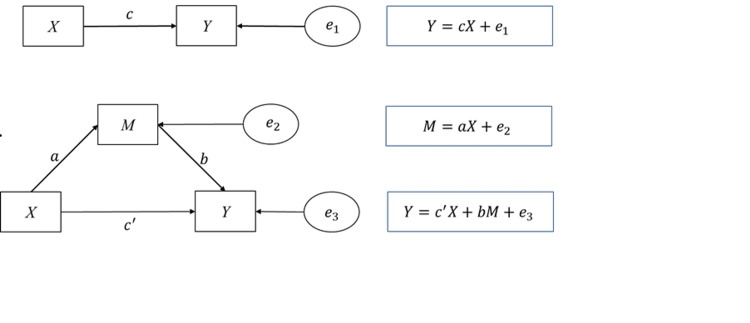
Intermediation effect flowchart

We employed general descriptive statistics to depict the sociodemographic characteristics of the sample, utilizing frequencies and percentages for categorical variables and means and standard deviations for continuous variables. To assess the binary correlation among healthy lifestyles, psychological resilience, and depressive symptoms, we conducted a Pearson correlation analysis. This study used stepwise regression analysis to explore the impact of (1) healthy lifestyles on depressive symptoms, (2) healthy lifestyles on psychological resilience, and (3) the influence of healthy lifestyles on depressive symptoms while controlling for psychological resilience. Control variables were introduced into each regression model to enhance the precision of the estimates. Furthermore, we applied the Sobel test and Bootstrap test to validate the robustness of the mediating effect and the proportions of effects. *X*, *Y*, and *M* in the path of mediated effects test denote the independent, dependent, and mediating variables, respectively. Here, *c* indicates the total effect, *c* denotes the direct effect value, and *a* and *b* denote the values in the intermediate effect process. *e* denote regression residuals (Figure 1).

## Results

Descriptive statistical results

A total of 9448 participants (46.06% male and 53.94% female) were included in this study. The age range was from 65 to 117 years, with a mean age of 83.16 years (SD = 11.29). The average years of education were 3.75 (SD = 4.40). Most of the participants were Han Chinese (94.54%), from rural areas (68.82%), and of common socioeconomic status (69.9%). Overall, older people self-reported their health status as good. In terms of marriage, less than half were married (45.4%). In terms of living arrangements, the majority of older people live with their families (83.62). In addition, the mean values for healthy lifestyles, psychological resilience, and depressive symptoms were 2.26, 19.49, and 7.34, respectively. The detailed results are presented in Table 1.

**Table 1:** Descriptive analysis of the demographic characteristics of the elderly in China

Variable	Frequency (n%) / Mean (SD)
Healthy lifestyles	2.26 (0.87)
Psychological resilience	19.49 (3.06)
Depressive symptoms	7.34 (4.78)
Age	83.16 (11.29)
Years of education	3.75 (4.40)
Gender	
Male	4352 (46.06)
Female	5096 (53.94)
Ethnic	
Han	8932 (94.54)
Non-Han	516 (5.46)
Household registration	
Rural	6502 (68.82)
Urban	2946(31.18)
Marriage	
Married	4289 (45.4)
Unmarried, widowed, divorced, or separated	5159 (54.6)
Living arrangements	
Living alone	1548 (16.38)
Not living alone	7900 (83.62)
Socio-economic status	
Poor	109 (1.15)
Relatively poor	794 (8.40)
Common	6604 (69.9)
Relatively rich	1670 (17.68)
Rich	271 (2.87)
Self-reported health	
Very bad	90 (0.95)
Relatively bad	1167 (12.35)
Normal	3643 (38.56)
Relatively good	3352 (35.48)
Very good	1196 (12.66)

Pearson correlation analysis results

Table 2 illustrates the correlation outcomes among the study variables. The results showed pairwise correlations involving healthy lifestyles, psychological resilience, and depressive symptoms. Specifically, a negative correlation was observed between healthy lifestyles and depressive symptoms (r = -0.102, p = 0.000), while a positive correlation emerged between healthy lifestyles and psychological resilience (r = 0.102, p =0.000). Furthermore, psychological resilience demonstrated a negative correlation with depressive symptoms (r = -0.768, p = 0.000).

**Table 2:** Results of Spearman's correlation analysis of the study sample

Variable	Healthy Lifestyles	Psychological resilience	Depressive symptoms
Healthy lifestyles	1		
Psychological resilience	0.102***	1	
Depressive symptoms	-0.102***	-0.768***	1
Note: *** p < 0.01.			

Results of regression analysis

We employed stepwise regression to examine the impact of healthy lifestyles on depressive symptoms, considering both the direct effect and the mediated effect with the inclusion of a mediator variable while controlling for relevant variables (Table 3). Adding the independent and control variables to model 1, the results showed a negative effect of healthy lifestyles and depressive symptoms (β = -0.310, 95% CI: -0.405, -0.215). Adding the independent and control variables to model 2 revealed a positive effect of healthy lifestyles and psychological resilience (β = 0.137, 95% CI: 0.071, 0.203). Adding the independent, mediator, and control variables simultaneously to model 3 showed that both healthy lifestyles and psychological resilience had a negative effect on depressive symptoms, with regression coefficient values of -0.171 (95% CI: -0.240, -0.102) and -1.014 (95% CI: -1.037, -0.990), respectively. The results of the stepwise regression supported hypotheses one, two, and three. Detailed results can be found in Table 3.

**Table 3:** Results of stepwise regression analysis of the main variables of the study sample * p < 0.1; ** p < 0.05; *** p < 0.01

Variable	Model 1 depressive symptoms		Model 2 psychological resilience		Model 3 depressive symptoms	
	Beta	(95% CI)	Beta	(95% CI)	Beta	(95% CI)
Healthy lifestyles	-0.310^***^	(-0.405, -0.215)	0.137^***^	(0.071, 0.203)	-0.171^***^	(-0.240, -0.102)
Psychological resilience					-1.014^***^	(-1.037, -0.990)
Age	0.023^***^	(0.014, 0.031)	-0.020^***^	(-0.026, -0.014)	0.002	(-0.004, 0.008)
Gender	-0.512^***^	(-0.686, -0.337)	0.327^***^	(0.204, 0.450)	-0.180^***^	(-0.306, -0.054)
Ethnic	-0.038	(-0.368, 0.291)	0.697^***^	(0.480, 0.915)	0.668^***^	(0.414, 0.923)
Household registration	0.071	(-0.131, 0.273)	0.282^***^	(0.143, 0.420)	0.357^***^	(0.215, 0.499)
Marriage	-0.411^***^	(-0.616, -0.205)	0.510^***^	(0.366, 0.654)	0.106	(-0.042, 0.254)
Years of education	-0.018	(-0.041, 0.005)	0.046^***^	(0.030, 0.062)	0.029^***^	(0.013, 0.045)
Socio-economic status	-0.991^***^	(-1.133, -0.849)	0.458^***^	(0.364, 0.552)	-0.527^***^	(-0.626, -0.427)
Living arrangements	-0.551^***^	(-0.788, -0.314)	-0.003	(-0.165, 0.158)	-0.554^***^	(-0.717, -0.391)
Self-reported health	-2.095^***^	(-2.192, -1.997)	1.301^***^	(1.235, 1.368)	-0.775^***^	(-0.847, -0.704)
Constant	17.484^***^	(16.516, 18.452)	13.600^***^	(12.950, 14.249)	31.268^***^	(30.497, 32.040)
Observations	9448		9448		9448	
R-squared	0.258		0.235		0.624	

Sobel and Bootstrap mediation effect tests

To further clarify the mediating role of psychological resilience in the relationship between healthy lifestyles and depressive symptoms in older adults, we conducted a robustness test using Sobel's test and Bootstrap method (replicated 1000 times; Table 4). The outcomes indicated that healthy lifestyles directly affect depressive symptoms with a direct effect value of -0.171 (p = 0.000, 95 CI: -0.243, -0.102). Additionally, healthy lifestyles indirectly affected depressive symptoms through psychological resilience with an indirect effect value of -0.139 (p = 0.000, 95 CI: -0.203, -0.073). Importantly, none of the 95% confidence interval ranges contained 0, suggesting that psychological resilience plays a partially mediating role in the influence of healthy lifestyles on depressive symptoms in older adults, thereby validating hypothesis four.

**Table 4:** Results of Sobel and Bootstrap tests for the study sample

Paths	Observed coefficient	Effect value as a percentage (%)	Sobel test			Bootstrap test		
			SE	Z	p	Bias	SE	95% CI
Indirect effect	-0.139	44.8	0.035	-3.969	0.000	0.002	0.034	-0.203, -0.073
Direct effect	-0.171	55.2	0.035	-4.83	0.000	-0.000	0.036	-0.243, -0.102
Total effect	-0.31		0.05	-6.231	0.000	0.001	0.048	-0.408, -0.212

## Discussion

Using data from the China Geriatric Health Survey, this study examined the relationship among healthy lifestyles, psychological resilience, and depressive symptoms in older adults. The results reveal a pairwise correlation involving healthy lifestyles, psychological resilience, and depressive symptoms. Importantly, psychological resilience serves as a partial mediator in the relationship between healthy lifestyles and depressive symptoms among older adults. The direct and indirect effects of healthy lifestyles on depressive symptoms accounted for 55.2% and 44.8%, respectively. Sobel and Bootstrap tests proved the robustness of the results of the underlying analyses. In summary, healthy lifestyles not only exert a direct influence on depressive symptoms but also operate indirectly through psychological resilience.

The results of this study showed that healthy lifestyles had a positive predictive effect on the prevention of depressive symptoms in Chinese older adults, which is consistent with previous studies. Dietary diversity, as identified in a study on the diets of older adults, was associated with a reduced risk of depressive symptoms. Varied foods supply essential substances and micronutrients regulating mood, such as folate and dietary fiber in vegetables, tryptophan and vitamin D in eggs, and zinc in beans [42]. These elements play a role in mood mediation by participating in the synthesis and metabolism of neurotransmitters, reducing inflammation, and stimulating the stress system [43]. Additionally, physical activity has been shown to effectively intervene and alleviate depressive symptoms through brain-derived neuromolecular mechanisms [44]. Notably, older adults who smoke and consume alcohol are more prone to depressive symptoms than non-smokers and non-drinkers [45]. Research indicates that nicotine can contribute to depressive symptoms by inducing physiological responses associated with anxiety and stress [46]. Excessive alcohol consumption may lead to structural changes in brain regions linked to depressive symptoms, including a smaller hippocampus volume, thinning of the medial orbitofrontal cortex, and thinning of the rostral anterior cingulate cortex [47]. Therefore, embracing a diverse range of healthy lifestyles may mitigate the risk of depressive symptoms in older Chinese adults, particularly considering life course disadvantages [48].

The study reveals that the healthy lifestyles of older adults exert a positive influence on psychological resilience, similar to previous studies. Adopting a positive psychology perspective, healthy lifestyles contribute enhanced emotional value to the daily lives of older adults, fostering improved psychological resilience [49]. Notably, physical activity demonstrates associations with heightened psychological resilience, reduced depressive symptoms, and fewer negative emotions [50]. Those engaging in appropriate physical activity are anticipated to exhibit elevated psychological resilience [51]. From a biological standpoint, healthy lifestyles among older adults can impact psychological resilience by modulating the central nervous system [52]. Research indicates an association between psychological resilience and lower cortisol levels [53], with cortisol secretion strongly linked to diet quality [54]. Furthermore, psychological resilience correlates with sleep, body weight, and dietary patterns [55-57]. Although there is less research evidence on healthy lifestyles and psychological resilience, our findings provide evidence for the effect of healthy lifestyles on psychological resilience. Based on this, we advocate for older adults to maintain healthy lifestyles, fostering enhanced psychological resilience and thereby promoting mental health.

Our study provides robust evidence supporting psychological resilience as a protective factor against depressive symptoms in older adults, consistent with prior research. Higher perceived psychological resilience plays a mediating role between social support and depressive symptoms in older adults, and cultivating psychological resilience may reduce the adverse effects of lower social support on depressive symptoms in this demographic [58]. Furthermore, superior psychological resilience may also play a potential buffering role in adult depression [59]. Individuals with heightened psychological resilience tend to harbor positive cognitive schemas and exhibit effective emotion regulation, contributing to stress alleviation in daily life [60]. In times of adversity, psychological resilience functions to attenuate the repercussions of negative events on the mental health of older adults. This attenuation is achieved through the promotion of positive cognitive styles, adaptive behaviors, strong social interactions, and neural underpinnings [61]. It has been demonstrated that those individuals who are more psychologically resilient possess greater stress resilience due to less coupling to negative events [62]. From a neurobiological perspective, psychological resilience can respond to daily stress by modulating multiple neural circuits [63]. For instance, positive behaviors in response to stress may involve transient activation of the hypothalamic-pituitary-adrenal axis [64] or norepinephrine [65]. Thus, cultivating enhanced psychological resilience proves advantageous in preventing the onset of depressive symptoms in older adults.

The results indicate that psychological resilience partially mediates the connection between healthy lifestyles and depressive symptoms, with the mediating effect accounting for 44.8% of the total effect. Adopting healthy lifestyles among older adults may heighten individual psychological resilience, consequently diminishing the likelihood of depressive symptoms. Healthy lifestyles contribute to the development of optimistic attitudes and positive emotions, resulting in greater tolerance and higher emotional resilience in the face of stress in older adults [66]. This resilience makes it easier for older adults to extricate themselves from negative emotions, thereby serving to prevent depressive symptoms. For example, the Mediterranean diet contributes to the regulation of cortisol, reduces levels of oxidative stress, and engages in mood regulation, which in turn is beneficial for mental health [67]. Additionally, regular physical activity correlates with higher psychological resilience and the amelioration of depressive symptoms through psychological resilience [68]. Similarly, psychological resilience can potentially protect against smoking-induced related trauma, thereby maintaining mental health [69]. Conversely, excessive drinking behaviors may lower psychological resilience in older adults, rendering them less capable of emotion regulation under stress and heightening the risk of depression [70].

Our findings elucidate the associations among healthy lifestyles, psychological resilience, and depressive symptoms, highlighting the mediating role of psychological resilience in the link between healthy lifestyles and depressive symptoms. These results offer insights for enhancing the mental well-being of older adults and facilitating active aging. Consequently, mental health policies and practices should integrate considerations of individual lifestyles and psychological resilience to formulate comprehensive interventions for depressive symptoms in older adults. Governments and policymakers can advance these efforts by instituting holistic health promotion programs that encompass initiatives for healthy lifestyle promotion, psychological resilience development, and health education and advocacy.

The interpretation of the study results necessitates consideration of several limitations. Firstly, the cross-sectional design used in this study does not allow for the determination of exact causal relationships between variables. To address this limitation, future studies could implement randomized controlled trials utilizing longitudinal data to substantiate causal connections. Secondly, the data used in this study were collected through a retrospective survey, which may introduce reporting bias. Finally, although several covariates were controlled for in this study, potential confounders may still be present, introducing endogeneity issues. In addition, there are limitations to our research design: mediating effects can only explain correlations between variables, not prove causality.

## Conclusions

This study explored the relationship between healthy lifestyles, psychological resilience, and depressive symptoms in older adults, using representative data from China. Findings suggest that healthy lifestyles are associated with a decreased risk of depressive symptoms in older adults. Furthermore, psychological resilience was identified as a partial mediator in the relationship between healthy lifestyles and depressive symptoms. Our research contributes to the understanding of the impact of integrated healthy lifestyles on depressive symptoms in older adults, underscoring the crucial role of psychological resilience. These findings carry significant implications for the development of mental health interventions and policies aimed at promoting healthy aging among older adults. Consequently, we propose that the government and relevant policymakers can enhance mental health in older adults by implementing comprehensive measures, including promoting healthy lifestyle education, offering psychological support services, and fostering a conducive environment.
